# Oroclines in the Central Asian Orogenic Belt

**DOI:** 10.1093/nsr/nwac243

**Published:** 2022-10-31

**Authors:** Yongjiang Liu, Wenjiao Xiao, Yongfei Ma, Sanzhong Li, A Yu Peskov, Zhaoxu Chen, Tong Zhou, Qingbin Guan

**Affiliations:** Frontiers Science Center for Deep Ocean Multispheres and Earth System, Key Lab of Submarine Geoscience and Prospecting Techniques, College of Marine Geosciences, Ocean University of China, China; Laboratory for Marine Mineral Resources, Qingdao National Laboratory for Marine Science and Technology, China; National Key Laboratory of Ecological Security and Sustainable Development in Arid Areas, Xinjiang Research Center for Mineral Resources, Xinjiang Institute of Ecology and Geography, Chinese Academy of Sciences, China; College of Earth Sciences, Institute of Disaster Prevention, China; Frontiers Science Center for Deep Ocean Multispheres and Earth System, Key Lab of Submarine Geoscience and Prospecting Techniques, College of Marine Geosciences, Ocean University of China, China; Laboratory for Marine Mineral Resources, Qingdao National Laboratory for Marine Science and Technology, China; Kosygin Institute of Tectonics and Geophysics, Far East Branch, Russian Academy of Sciences, Russia; Frontiers Science Center for Deep Ocean Multispheres and Earth System, Key Lab of Submarine Geoscience and Prospecting Techniques, College of Marine Geosciences, Ocean University of China, China; Laboratory for Marine Mineral Resources, Qingdao National Laboratory for Marine Science and Technology, China; Frontiers Science Center for Deep Ocean Multispheres and Earth System, Key Lab of Submarine Geoscience and Prospecting Techniques, College of Marine Geosciences, Ocean University of China, China; Laboratory for Marine Mineral Resources, Qingdao National Laboratory for Marine Science and Technology, China; Frontiers Science Center for Deep Ocean Multispheres and Earth System, Key Lab of Submarine Geoscience and Prospecting Techniques, College of Marine Geosciences, Ocean University of China, China; Laboratory for Marine Mineral Resources, Qingdao National Laboratory for Marine Science and Technology, China

The term ‘orocline’ was first proposed by Carey [1] to describe the structure as ‘an orogenic system which has been flexed in plane to a horse-shoe or elbow shape’; it usually emphasizes that a previous straight mountain belt or tectonic units have been bended horizontally. Nowadays the orocline has been used to describe any curved mountain belt regardless of its original shape [2]. Oroclines were often formed by the rollback of subducting slab, buckling and indentation that resulted from convergence along active and intra-oceanic margins [2] or by compression parallel to the orogenic belt [3].

The CAOB, the southern part of which is also called the Altaids [4], lies between the Siberia craton to the north and the North China-Tarim craton to the south (Fig. 1a). It is one of the largest accretionary orogenic belts in the world and characterized both by the amalgamation of multiple terranes and oroclinal bending, in which the Kazakhstan, Mongolian and NE China oroclines from west to east (Fig. 1a) were produced by the complicated accretionary processes of many intra-oceanic arcs and micro-blocks, related to the subduction rollback of the Paleo-Asian Ocean and compression due to the rotation and convergence of the neighboring continental blocks or surround subduction [2,5]. The Kazakhstan orocline in the western CAOB consists of old basement, Ordovician arc complex, the Devonian Volcanic Belt and the Late Devonian to Carboniferous Balkhash-Yili arc, which was first formed as a nearly straight accretionary orogen in the early-middle Devonian and later buckled by compression due to the rotation of the Siberian craton [2,6,7] during the assembly of the Siberian and Tarim cratons [8] (Fig. 1b).

The Mongolian orocline in the middle CAOB consists of the Tuva-Mongol micro-continental ribbon and Paleozoic accretionary terranes, arcs and sediments, which were produced by the bending of a pre-Late Carboniferous linear accretionary orogenic belt due to the rotation of its southern limb related to the rotation and amalgamation of the Siberian and North China cratons in the late stage [2,9,10] (Fig. 1c). The NE China orocline (called the Hingan orocline by Yakubchuk [11] but with different construction) consists of a curved Erguna-Jiamusi continent ribbon, early Paleozoic Xing’an-Zhangguancailing accretionary terranes and late Paleozoic Songliao accretionary terranes with some Precambrian micro-block relics in the core area [5] (Fig. 1d), which was formed dominantly by long-lived accretionary processes. The oroclinal bending was attributed to the rollback of the subducted Paleo-Asian oceanic slab during the most Paleozoic period [5] and its final bending was with the help of the convergent compression related to the westward subduction of the Paleo-Pacific Ocean and southeastward subduction of the Mongol-Okhotsk Ocean in the latest Paleozoic to Mesozoic time (Fig. 1d). The northwestern limb of the NE China orocline connects to the southern limb of the Mongolian orocline, which together compose ‘S’-shaped double oroclines (Fig. 1e).

Considering the different formation processes and curvature mechanisms, oroclines can be divided into two endmembers: the rollback orocline and the buckling orocline. The rollback orocline is being progressively bended by the rollback processes of subducted oceanic slab during the accretionary orogeny with a long-lived active margin and multiple intra-oceanic arcs (e.g. oroclines in the CAOB [2,5]).

The buckling orocline is generally formed by buckling of an originally straight or nearly straight orogenic belt, which formed in the first stage and was buckled in the second stage either by mountain-parallel/oblique compression (e.g. Cantabrian orocline [12]) or by mountain-perpendicular/oblique indentation (e.g. Adriatic Orocline [13], Peruvian and Bolivian oroclines [3]).

In fact, the two endmembers, rollback orocline and buckling orocline, may not exist absolutely in the natural situation. For example, the latest bending of the NE China orocline was somewhat with the help of regional convergent compression, while the first stage of the Kazakhstan and Mongolian oroclines were related to the accretionary process due to subduction retreat. Therefore, they all experienced the first stage of forming a nearly straight accretionary orogen and were then bent due to the rollback process and/or buckled due to compression in the second stage. All three oroclines in the CAOB were related to a long-lived subduction–accretion orogenic process. Therefore, the orocline structure plays a key role in the development of accretionary orogen architecture and contributes to the formation of a huge orogenic belt, such as the CAOB.

**Figure 1.**
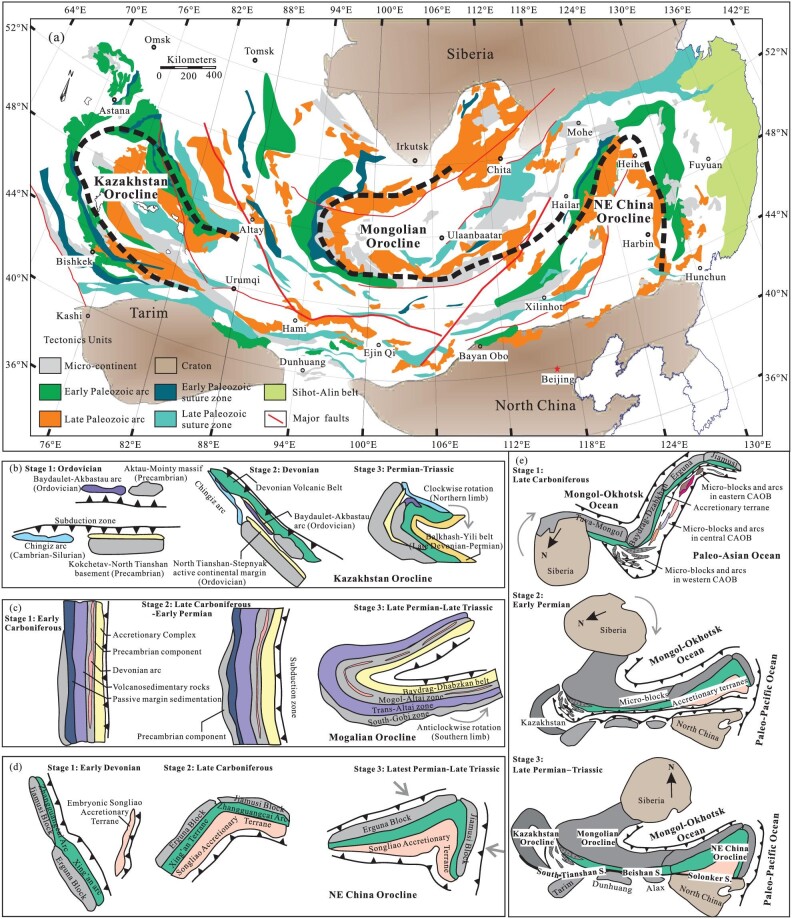
(a) Sketched geological map of the oroclines in CAOB. (b) Kazakhstan orocline formation models. (c) Mongolian orocline formation models. (d) NE China orocline formation model. (e) Spatial relationship of the oroclines in CAOB. Modified from Refs [2,5,7].

## References

[1] Carey S-W . Proc R Soc Tasmania Part-I 1955; 89: 255–88.

[2] Xiao W-J , Windley B, Sun S et al. Annu Rev Earth Planet Sci 2015; 43: 477–507. 10.1146/annurev-earth-060614-105254

[3] Johnston S-T , Weil A-B, Gutierrez-Alonso G. Geol Soc Am Bull 2013; 125: 643–63. 10.1130/B30765.1

[4] Şengör A-M-C , Natal’in B-A, Burtman V-S. Nature 1993; 364: 299–307. 10.1038/364299a0

[5] Liu Y-J , Li W-M, Ma Y-F et al. Earth-Sci Rev 2021; 221: 103808. 10.1016/j.earscirev.2021.103808

[6] Abrajevitch A , Van der voo R, Bazhenov M-L et al. Tectonophysics 2008; 455: 61–76. 10.1016/j.tecto.2008.05.006

[7] Xiao W-J , Brian F-W, Han C-M et al. Earth-Sci Rev 2018; 186: 94–128. 10.1016/j.earscirev.2017.09.020

[8] Li P-F , Sun M, Rosenbaum G et al. J Asian Earth Sci 2018; 153: 42–56. 10.1016/j.jseaes.2017.07.029

[9] Li P-F , Sun M, Narantsetseg T et al. Geol Soc Am Bull 2021; 134: 1994–2006. 10.1130/B36200.1

[10] Edel J-B , Schulmann K, Hanzl et al. J Asian Earth Sci 2014; 94: 157–71. 10.1016/j.jseaes.2014.07.039

[11] Yakubchuk A . J Asian Earth Sci 2004; 23: 761–79. 10.1016/j.jseaes.2004.01.006

[12] Shaw J , Johnston S-T, Gutiérrez-Alonso G. Lithosphere 2015; 7: 653–61. 10.1130/B36200.1

[13] Rosenbaum G . J Geodyn 2014; 82: 5–15. 10.1016/j.jog.2014.05.002

